# Robustness and Vulnerability of the Autoregulatory System That Maintains Nuclear TDP-43 Levels: A Trade-off Hypothesis for ALS Pathology Based on *in Silico* Data

**DOI:** 10.3389/fnins.2018.00028

**Published:** 2018-02-01

**Authors:** Akihiro Sugai, Taisuke Kato, Akihide Koyama, Yuka Koike, Sou Kasahara, Takuya Konno, Tomohiko Ishihara, Osamu Onodera

**Affiliations:** ^1^Department of Neurology, Clinical Neuroscience Branch, Brain Research Institute, Niigata University, Niigata, Japan; ^2^Department of System Pathology for Neurological Disorders, Brain Science Branch, Center for Bioresource-Based Research, Brain Research Institute, Niigata University, Niigata, Japan; ^3^Division of Legal Medicine, Graduate School of Medicine and Dental Science, Niigata University, Niigata, Japan; ^4^Department of Molecular Neuroscience, Resource Branch for Brain Disease Research, Center for Bioresource-based Research, Brain Research Institute, Niigata University, Niigata, Japan

**Keywords:** amyotrophic lateral sclerosis, TDP-43, *TARDBP*, autoregulation, robustness, systems biology

## Abstract

Abnormal accumulation of TAR DNA-binding protein 43 (TDP-43) in the cytoplasm and its disappearance from the nucleus are pathological features of amyotrophic lateral sclerosis and frontotemporal dementia (ALS/FTD) and are directly involved in the pathogenesis of these conditions. TDP-43 is an essential nuclear protein that readily aggregates in a concentration-dependent manner. Therefore, cells must strictly maintain an appropriate amount of nuclear TDP-43. In one relevant maintenance mechanism, TDP-43 binds to its pre-mRNA and promotes alternative splicing, resulting in mRNA degradation via nonsense-mediated mRNA decay. The level of nuclear TDP-43 is tightly regulated by these mechanisms, which control the amount of mRNA that may be translated. Based on the results of previous experiments, we developed an *in silico* model that mimics the intracellular dynamics of TDP-43 and examined TDP-43 metabolism under various conditions. We discovered an inherent trade-off in this mechanism between transcriptional redundancy, which maintains the robustness of TDP-43 metabolism, and vulnerability to specific interfering factors. These factors include an increased tendency of TDP-43 to aggregate, impaired nuclear-cytoplasmic TDP-43 transport, and a decreased efficiency of degrading abnormal proteins, all of which are functional abnormalities related to the gene that causes familial ALS/FTD. When these conditions continue at a certain intensity, the vulnerability of the autoregulatory machinery becomes apparent over time, and transcriptional redundancy enters a vicious cycle that ultimately results in TDP-43 pathology. The results obtained using this *in silico* model reveal the difference in TDP-43 metabolism between normal and disease states. Furthermore, using this model, we simulated the effect of a decrease in TDP-43 transcription and found that this decrease improved TDP-43 pathology and suppressed the abnormal propagation of TDP-43. Therefore, we propose a potential therapeutic strategy to suppress transcriptional redundancy, which is the driving force of the pathological condition caused by the specific factors described above, in patients with ALS presenting with TDP-43 pathology. An ALS animal model exhibiting TDP-43 pathology without overexpression of exogenous TDP-43 should be developed to investigate the effect of alleviating the transcriptional redundancy of *TARDBP*.

## Introduction

Amyotrophic lateral sclerosis (ALS) is a devastating neurological disease characterized by the degeneration of upper and lower motor neurons. ALS leads to death within 2–5 years as a result of muscle weakness, including respiratory dysfunction. Up to 50% of patients with ALS develop cognitive and behavioral abnormalities, and approximately 13% of patients with ALS present with concomitant behaviorally variant frontotemporal dementia (FTD) (van Es et al., 2017). These two neurodegenerative diseases have a common pathological background caused by the abnormal accumulation of TAR DNA-binding protein 43 (TDP-43). This feature occurs in nearly all patients with ALS and in up to 50% of patients with FTD (Arai et al., 2006; Neumann et al., 2006). The accumulation of TDP-43 correlates with the spread of neurodegeneration, suggesting that pathological conditions progress between cells through the propagation of abnormal TDP-43 accumulation (Polymenidou and Cleveland, 2011; Braak et al., 2013; Brettschneider et al., 2013; Nonaka et al., 2013). A mutation in the *TARDBP* gene, which encodes TDP-43, is present in 1–5% of patients with familial ALS, and these patients exhibit TDP-43 pathology similar to individuals with sporadic ALS. The same TDP-43 pathology has also been identified in patients with mutations in many ALS-causative genes, including hexanucleotide repeat expansions in *C9ORF72*, a major genetic cause of ALS/FTD (van Es et al., 2017). Based on these findings, abnormalities in TDP-43 are directly involved in the pathogenesis of ALS/FTD.

TDP-43 is a nuclear protein that moves between the nucleus and cytoplasm through a nuclear localization signal and a nuclear export signal, respectively (Ayala et al., 2008; Winton et al., 2008). TDP-43 also contains RNA recognition motifs, binds to RNAs, and affects a wide range of RNA metabolic processes, including pre-mRNA processing, microRNA regulation, and the control of long noncoding RNA and mRNA transport (Ling et al., 2013; Ratti and Buratti, 2016). In addition, TDP-43 contains a prion-like domain and regulates the formation and dynamics of stress granules (McDonald et al., 2011; Sun and Chakrabartty, 2017). Stress granules are membraneless organelles that are involved in the transient and reversible sequestration of undesirable transcripts. RNA-binding proteins that contain prion-like domains undergo liquid-liquid phase separation, which underlies the formation of these membraneless organelles (Molliex et al., 2015; Sun and Chakrabartty, 2017). The properties of TDP-43, an aggregation-prone protein, contribute to stress granule formation.

As TDP-43 is extensively involved in RNA metabolism and tends to aggregate, cell function and survival depend on the strict control of TDP-43 levels (Lee et al., 2012; Ling et al., 2013). TDP-43 knockout mice display embryonic lethality (Kraemer et al., 2010; Sephton et al., 2010). Adult mice in which TDP-43 is conditionally deleted also die shortly after the loss of TDP-43 expression (Chiang et al., 2010), and the motor neuron-specific loss of TDP-43 leads to age-dependent motor neuron degeneration (Wu et al., 2012; Iguchi et al., 2013). Moreover, TDP-43 overexpression leads to neuronal degeneration and death in a dose-dependent manner (McGoldrick et al., 2013). However, TDP-43 expression is increased in the central nervous system, cerebrospinal fluid, and plasma of patients with ALS (Kasai et al., 2009; Swarup et al., 2011b; Verstraete et al., 2012; Iguchi et al., 2016). Additionally, in patients with ALS/FTD, TDP-43 diminishes or disappears from the nucleus and forms inclusion bodies with fragmented products in the cytoplasm (Arai et al., 2006; Neumann et al., 2006). Currently, the functional losses caused by the disappearance of nuclear TDP-43 and the toxicity caused by the formation of aggregates in the cytoplasm are both postulated to contribute to the pathogenesis of ALS/FTD (Lee et al., 2012; Ling et al., 2013).

**Figure 1:**
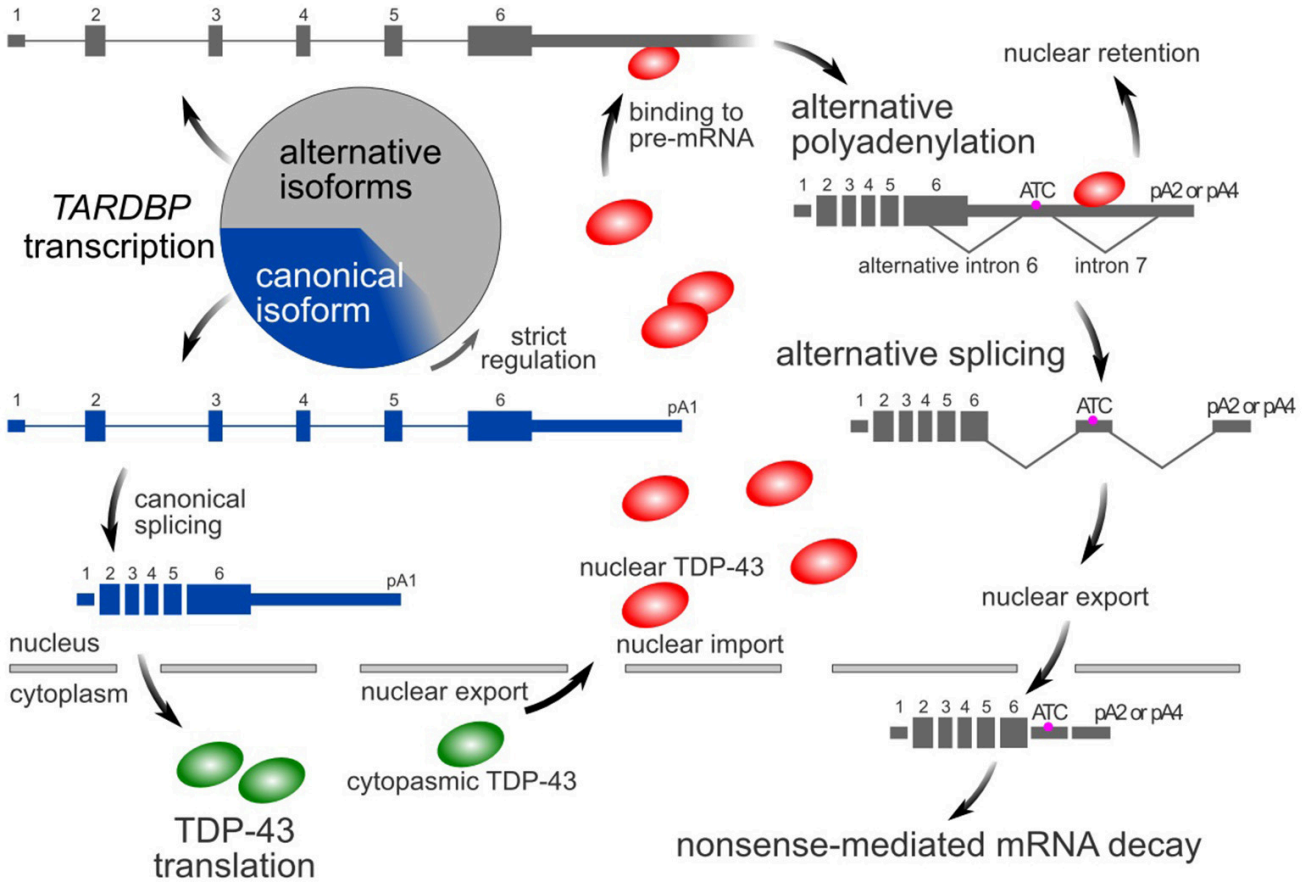
TDP-43 autoregulatory mechanism. In the canonical isoform (blue box) of the *TARDBP* mRNA, pA1 is used as a poly A site, and introns 6 and 7 are not spliced. In its alternative isoforms (gray boxes), pA2 or pA4 is used as a poly A site, and introns 6 and 7 are spliced. The amount of nuclear TDP-43 determines the ratio of these isoforms. Because the alternative isoforms are ultimately destined to undergo rapid degradation via nonsense-mediated mRNA decay, only a small amount of these forms is typically detected. However, these alternatively spliced isoforms comprise more than half of the transcripts. The circle represents the total amount of transcribed *TARDBP* mRNA.

Similar to many RNA-binding proteins (Huelga et al., 2012; Zhou et al., 2013), TDP-43 autoregulates its own expression through a negative feedback mechanism that depends on nuclear TDP-43 (Ayala et al., 2011; Polymenidou et al., 2011). The *TARDBP* pre-mRNA contains multiple alternative introns and polyadenylation signals in its last exon (Avendaño-Vázquez et al., 2012; Koyama et al., 2016). In the nucleus, TDP-43 binds to the 3′-UTR of its pre-mRNA, resulting in the use of distal poly A sites (alternative polyadenylation) (Avendaño-Vázquez et al., 2012; Koyama et al., 2016). Multiple alternative introns are then consecutively spliced (Koyama et al., 2016). The resulting isoform has an additional termination codon located more than 50 nucleotides upstream of the final exon junction complex. These alternatively spliced variants are susceptible to nonsense-mediated mRNA decay (Polymenidou et al., 2011; Koyama et al., 2016). However, some RNAs avoid alternative splicing, despite the use of distal poly A sites. These RNAs tend to localize in the nucleus and therefore do not contribute to translation in the cytoplasm (Koyama et al., 2016). Thus, by processing its pre-mRNA, the amount of nuclear TDP-43 precisely regulates the intracytoplasmic *TARDBP* mRNA level (Figure 1).

Notably, major ALS-related RNA-binding proteins with a prion-like domain have an autoregulatory mechanism (Le Guiner et al., 2001; Zhou et al., 2013; Suzuki and Matsuoka, 2017). Several ALS-causing mutations in *FUS* and *hnRNPA1* disrupt the nuclear localization sequence and thus increase the amount of these factors in the cytoplasm (Dormann et al., 2010; Liu et al., 2016). When nuclear-cytoplasmic transport is impaired, the autoregulatory mechanism enhances mRNA expression by reducing the nuclear protein level, leading to a further increase in the amount of the protein in the cytoplasm (Zhou et al., 2013). ALS-related RNA-binding proteins such as FUS, hnRNPA1, TIA1, and TDP-43 have been reported to undergo liquid-liquid phase separation through a mechanism that involves a prion-like domain (Molliex et al., 2015; Gopal et al., 2017; Mackenzie et al., 2017). This liquid-liquid phase transition strongly depends on the local concentrations of RNA-binding proteins (Molliex et al., 2015; Boeynaems et al., 2016). Therefore, the entanglement of the mechanisms of autoregulation with the mechanisms of RNA granule formation involving these RNA-binding proteins may contribute to the pathogenesis of ALS.

Here, we created an *in silico* model mimicking the intracellular dynamics of TDP-43 to determine the vulnerability of the mechanism regulating TDP-43 levels in the nucleus. Using this model, we show that robustness in the maintenance of nuclear TDP-43 by autoregulation conversely results in a decrease in nuclear TDP-43 and enhances aggregate accumulation through a pathological spiral driven by redundant transcription under several specific conditions. The role of TDP-43 in the formation of membraneless organelles and the mechanism that strictly controls the amount of nuclear TDP-43 through transcriptional redundancy may conflict in motor neurons in patients with ALS. Based on this hypothesis, we focus on the role of *TARDBP* transcriptional redundancy in the pathogenesis of ALS and propose a treatment strategy to control abnormal TDP-43 metabolism in patients with ALS.

## Establishing the *in silico* model of TDP-43 dynamics in cells

What is the weak point in the mechanism that controls TDP-43 levels? Moreover, how do pathological abnormalities in TDP-43, including its cytoplasmic translocation, fragmentation, and aggregation, occur? We applied an *in silico* model that simulates the intracellular dynamics of TDP-43 in CellDesigner (v4.4; Systems Biology Institute, Tokyo, Japan), a structured diagram editor used to draw gene-regulatory and biochemical networks (Funahashi et al., 2003), to investigate these issues. For our research purposes, we presumed that a description of the absolute quantity and absolute timing of each element and its variation in the cell was unnecessary. Therefore, a relative amount and relative time were applied to descriptions of each element and its variation. In the normal state where no disturbance was applied, each element was assumed to be static without fluctuations. Each setting of the model was designed to maintain consistency with our experimental results as described below and with the findings reported in previous experiments. In addition, the settings of the model were adjusted to be consistent with the results from animal models described below, and finally the adequacy of the model was confirmed (Supplementary Tables 1–3, Data Sheet 1).

### Autoregulation

TDP-43 binds to its pre-mRNA in the nucleus to negatively control cytoplasmic mRNA levels (negative autoregulation; NAR). The *TARDBP* pre-mRNA is transcribed above the level required for normal conditions, and the extra mRNA is degraded via nonsense-mediated mRNA decay through alternative splicing (Figure 1). A decrease in TDP-43 protein and mRNA levels does not occur in the central nervous system of *TARDBP* heterozygous knockout mice (Kraemer et al., 2010; Sephton et al., 2010; Ricketts et al., 2014). Thus, the transcription of the *TARDBP* mRNA displays at least twice as much redundancy as the normal state and at least 50% of the *TARDBP* pre-mRNA is alternatively spliced. In addition, in the analysis of mouse embryonic stem (ES) cells expressing human TDP-43 cDNA under the control of the endogenous mouse *TARDBP* promoter, the amount of TDP-43 protein increases approximately 3-fold in the absence of the *TARDBP* 3′-UTR, which is essential for autoregulation (Stribl et al., 2014). Furthermore, following the silencing of endogenous TDP-43 with siRNAs in HEK293T cells, alternative splicing of the minigene containing the last exon of human *TARDBP* is markedly diminished, resulting in a 3- to 4-fold increase in the canonical mRNA level of the minigene (Koyama et al., 2016). Based on this result, 67–75% of the pre-mRNA of the human *TARDBP* minigene is alternatively spliced.

#### Alternative splicing of the *TARDBP* mRNA in the mouse cerebrum

**Figure 2:**
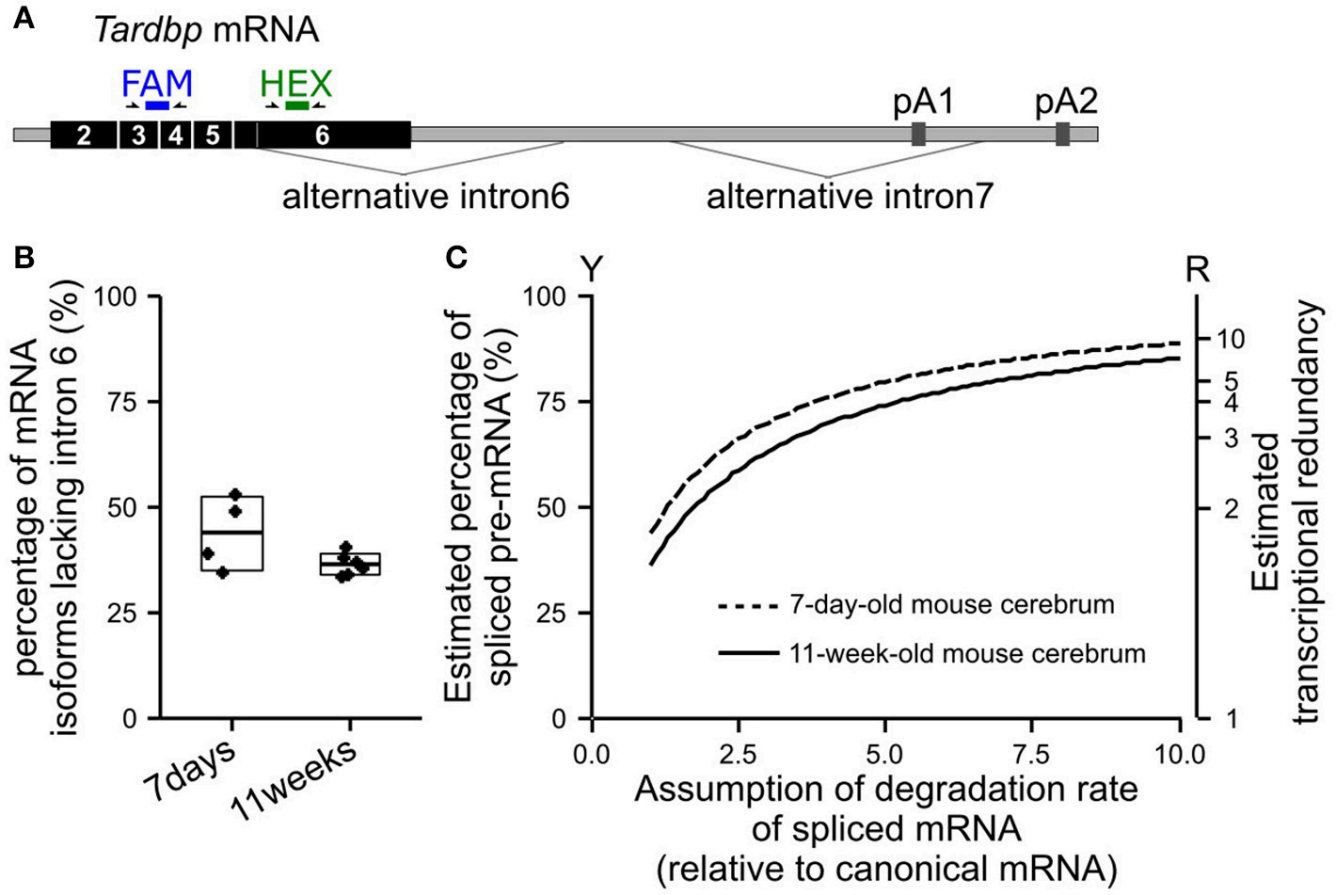
Estimated percentage of alternatively spliced pre-mRNAs in the mouse cerebrum. **(A)** The positions of the primers and probes used for Droplet Digital PCR to determine the percentage of isoforms lacking alternative intron 6 among all *TARDBP* mRNA isoforms are shown. The FAM probe detects all isoforms of the *TARDBP* mRNA, and the HEX probe detects isoforms that retain alternative intron 6. **(B)** The percentages of isoforms lacking alternative intron 6 in the cerebrum of 7-day-old and 11-week-old wild-type mice (C57BL/6NCrl) are shown. The upper and lower sides of the box indicate the standard deviation. The lines in the box indicate the average. **(C)** Estimated percentage of alternatively spliced pre-mRNAs among the total transcribed *TARDBP* pre-mRNA is shown; the degradation rate of the isoforms lacking alternative intron 6 relative to the canonical mRNA was estimated (horizontal axis). Transcriptional redundancy (R) is represented by the equation *R* = 100/(100-Y), where Y indicates the percentage of pre-mRNA that has undergone alternative splicing among all transcribed pre-mRNAs.

We extracted total RNA from the cerebrum of wild-type mice (C57BL/6NCrl) using NucleoSpin RNA II (TaKaRa Bio, Japan) to confirm the percentage of the *TARDBP* pre-mRNA that undergoes alternative splicing *in vivo*. The percentage of alternatively spliced isoforms among all isoforms of the *TARDBP* mRNA was examined using the Droplet Digital PCR method (Figure 2A). Since Droplet Digital PCR enables the absolute quantification of target RNAs, the expression ratio of each isoform was determined. The primers and probe sequences used for Droplet Digital PCR are shown in Supplemental Table 4. The average percentages of isoforms lacking alternative intron 6 of the *TARDBP* mRNA in the cerebrum of 7-day-old and 11-week-old mice were 44 and 36%, respectively (Figure 2B). For most of the mRNA in which alternative intron 6 is spliced, alternative intron 7 is also spliced, and this isoform undergoes degradation through nonsense-mediated mRNA decay (Koyama et al., 2016). The percentage of the *TARDBP* pre-mRNA that underwent alternative splicing was calculated by determining the rate of degradation of isoforms lacking the alternative intron 6. We were not able to accurately determine the rate of degradation of *TARDBP* mRNA isoforms that were alternatively spliced in the mouse cerebrum. However, when the degradation rate was assumed to be only 3–5 times greater than the rate of the canonical *TARDBP* mRNA, which may be lower than the general degradation rate due to nonsense-mediated mRNA decay (Tani et al., 2012), the percentage of alternatively spliced *TARDBP* pre-mRNA (Figure 2C; Y axis) was estimated to be 63% to 74% in the cerebrum of adult mice. After defining transcriptional redundancy as 100/(100-Y), where Y indicates the percentage of pre-mRNA that has undergone alternative splicing, the redundancy of *TARDBP* transcription in the adult mouse cerebrum was estimated to be 2.7–3.8 (Figure 2C; R axis). The results from the mouse cerebrum analysis are consistent with the findings from previous studies (Kraemer et al., 2010; Sephton et al., 2010; Ricketts et al., 2014; Stribl et al., 2014; Koyama et al., 2016).

Based on these experimental results and previous findings, we hypothesized that at least 50%, and potentially 60–80%, of the transcribed *TARDBP* RNA is degraded by nonsense-mediated mRNA decay. This transcriptional redundancy may be influenced by several conditions, including conditions within the tissue or cell (Kraemer et al., 2010; Sephton et al., 2010), environmental factors, and age (Figure 2B). Therefore, in our model, parameters representing the efficiency of autoregulation were initially determined by estimating that 65% of the transcribed RNA was degraded by nonsense-mediated mRNA decay. The rate at which the canonical mRNA was produced was approximated using the Hill function (Supplementary Table 1). In this function, the product of the pre-mRNA and the coefficient (k2) define the maximum amount of canonical mRNA expression. The initial amount of the pre-mRNA is a constant. The suppression coefficient (Knar) indicates the concentration of nuclear TDP-43 that suppresses the maximal production of canonical mRNA by 50%. Knar is assumed to depend on the affinity of TDP-43 for the TDP-43-binding region of the *TARDBP* 3′-UTR and represents the efficiency of the autoregulatory mechanism.

### Nuclear and cytoplasmic TDP-43 levels

TDP-43 is shuttled between the nucleus and cytoplasm (Ayala et al., 2008; Winton et al., 2008). Immunohistochemistry for TDP-43 revealed a cytoplasmic/nuclear ratio of TDP-43 of 0.05–0.2 under normal conditions (Matsukawa et al., 2016; Khosravi et al., 2017; Woo et al., 2017). Therefore, in our model, the ratio of cytoplasmic/nuclear TDP-43 was set to 0.15. The half-life of TDP-43 lacking a nuclear export signal is sufficiently longer than the half-life of TDP-43 lacking a nuclear localization signal (Watanabe et al., 2012). Therefore, in this model, the degradation rate for cytoplasmic TDP-43 was set to 5-times the degradation rate for nuclear TDP-43. In experiments with SH-SY5Y cells, the half-life of the TDP-43 protein is approximately 20 times the half-life of the mRNA (Scotter et al., 2014). Thus, in this model, the degradation rate of nuclear TDP-43 was set to 0.05 times the mRNA degradation rate.

### Aggregation and fragmentation

**Figure 3:**
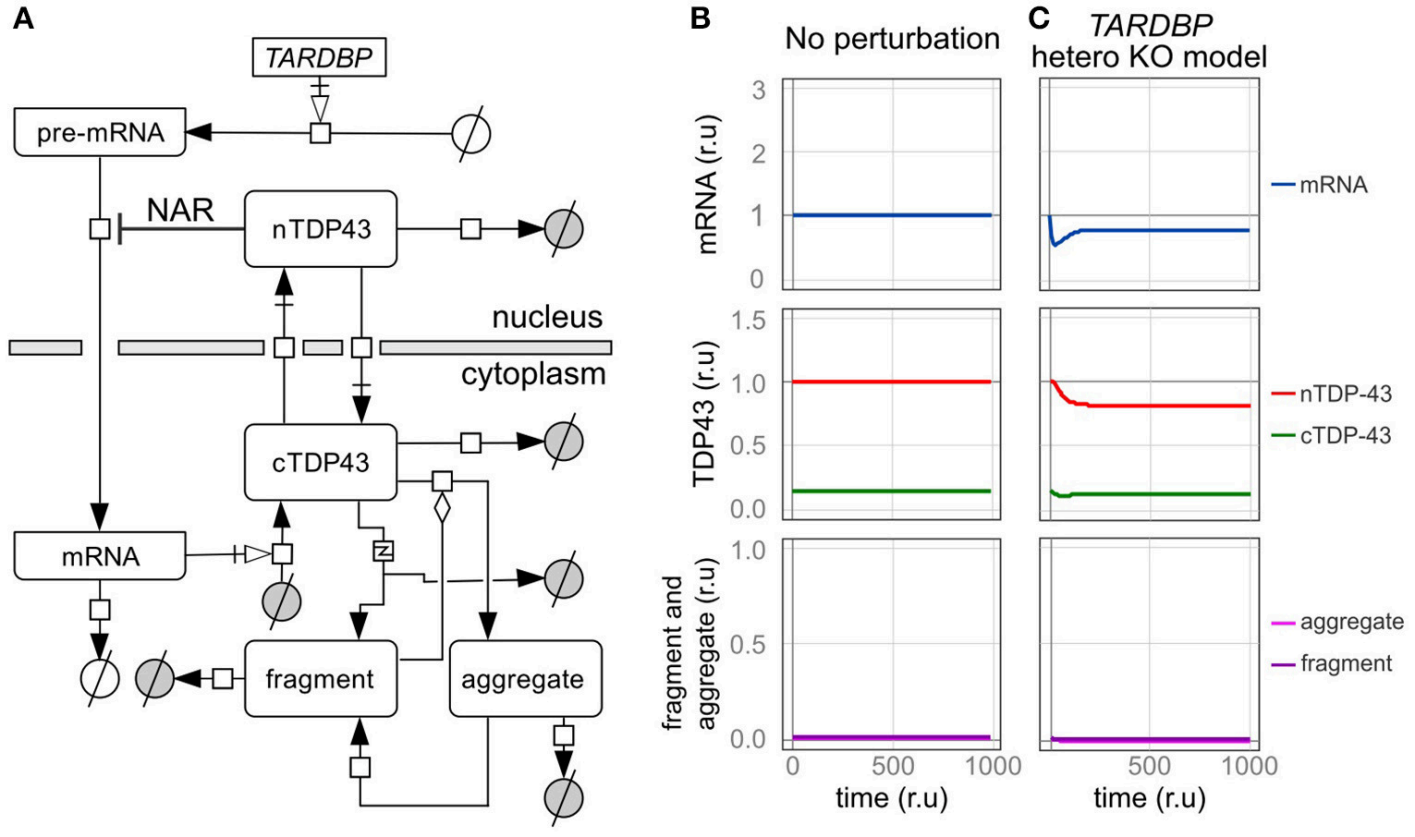
An *in silico* model of TDP-43 metabolism. **(A)** Graphic representation of TDP-43 metabolism in cells with TDP-43-negative autoregulation (NAR). nTDP43 and cTDP43 represent nuclear and cytoplasmic TDP-43, respectively. mRNA represents the canonical *TARDBP* mRNA that is translated in the cytoplasm. The gray and white circles represent amino acids and nucleic acids, respectively. The squares represent each reaction. Black arrows represent state transitions. White arrows with squares represent transcription or translation. The diamond represents modulation. The square including “z” represents truncation. **(B,C)** Changes in the level of each element are shown for a case in which no disturbance was added to the cell over time and **(B)** a case in which the amount of transcription was reduced by half **(C)**. The mRNA level is expressed relative to the initial level. Levels of nuclear TDP-43, cytoplasmic TDP-43, and fragmented and aggregated TDP-43 are expressed relative to the initial level of nuclear TDP-43 (r.u., relative unit).

The TDP-43 protein is intrinsically disordered and prone to aggregation because it contains a low-complexity prion-like domain at its C-terminus (Johnson et al., 2009; Budini et al., 2012). The TDP-43 concentration strongly affects this aggregation-prone property. TDP-43 accumulates in cytoplasmic stress granules, and the formation of these puncta is thought to be caused by a phenomenon referred to as the phase transition, which depends on the concentration of the protein in the cytoplasm (Molliex et al., 2015; Sun and Chakrabartty, 2017). Therefore, in our model, the rate of aggregate formation was defined based on the amount of TDP-43 in the cytoplasm (Figure 3A). Treatment of SH-SY5Y cells with the proteasome inhibitor MG132 induces the formation of aggregates and increases the amount of high molecular weight, urea-soluble TDP-43. Although the half-life of the 43 kDa TDP-43 protein in this cultured cell line is approximately 30 h, the level of urea-soluble TDP-43 remains slightly reduced 48 h after MG132 wash out (Scotter et al., 2014). Therefore, in this model, the degradation rate of aggregates was assumed to be 0.2 times the degradation rate of nuclear TDP-43.

TDP-43 fragmentation is a biochemical characteristic of ALS/FTD. Although TDP-43 fragmentation occurs under normal conditions, it increases in the presence of an excessive amount of TDP-43 (Hasegawa et al., 2008; Kabashi et al., 2008; Xu et al., 2010). In addition, an excess amount of fragmented TDP-43 products has been reported to induce and promote aggregation by trapping the normal TDP-43 protein, and this aggregate may be fragmented (Igaz et al., 2009; Nonaka et al., 2009; Furukawa et al., 2011). Therefore, in this model, the extent of TDP-43 fragmentation was based on the amount of cytoplasmic TDP-43, and the rate of aggregate formation was modified by the amount of accumulated fragmented TDP-43 (Figure 3A). Based on experiments using SH-SY5Y and HEK293 cells, the TDP-43 C-terminal fragment degrades more quickly than wild-type TDP-43, and its half-life was approximately one-third the half-life of wild-type TDP-43 (Scotter et al., 2014). Thus, in this model, the degradation rate of fragments was set to 3 times the degradation rate of cytoplasmic TDP-43. For both aggregated and fragmented TDP-43, the parameters related to the initial value, production efficiency, and decomposition efficiency were set based on the assumption that only a trace amount of fragments exist in the normal state (Figure 3B).

## Validation of the *in silico* model

### *TARDBP* heterozygous knockout model

We initially assessed a *TARDBP* heterozygous knockout model in which the transcription of *TARDBP* is reduced by half to determine whether our *in silico* model designed to investigate TDP-43 autoregulation produces results consistent with the findings from previous studies (Figure 3C). In this model, the level of transcription is reduced because only one allele is present, and in the level of the *TARDBP* mRNA is initially decreased. However, the level of *TARDBP* mRNA is restored by an autoregulatory mechanism and eventually stabilizes at 79% of the initial value. In addition, the amount of TDP-43 in the nucleus is not reduced by half and is maintained at 82% of the initial value. In experimental analyses of *TARDBP* heterozygous knockout mice, TDP-43 expression is not different from the level of the mRNA or protein observed in wild-type mice (Kraemer et al., 2010; Sephton et al., 2010; Ricketts et al., 2014). However, because the efficiency of the alternative splicing of *SORT1* and *PDP1* pre-mRNAs, which are target RNAs of TDP-43, is decreased, we suggest that TDP-43 expression is only slightly reduced, as the western blot results did not reveal differences (Ricketts et al., 2014). Thus, the results obtained using the dynamics established in this model display good agreement with the results of previous analyses of this *TARDBP* heterozygous knockout mouse line.

### *TARDBP* transgenic model

**Figure 4:**
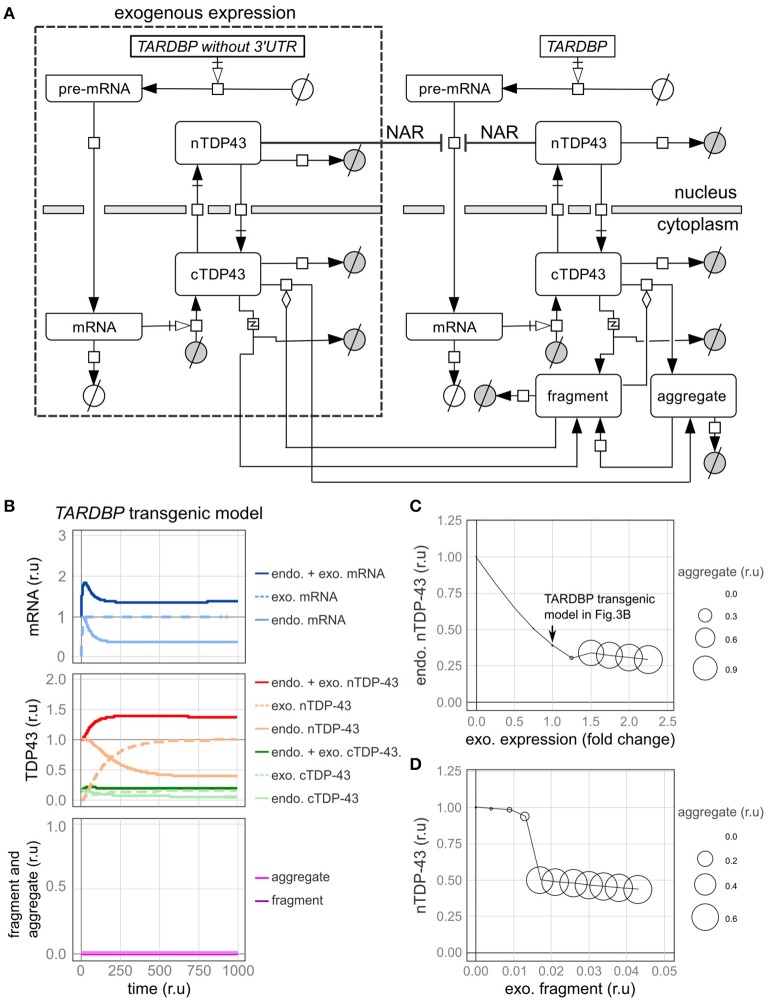
A model of transgenic *TARDBP* expression and a model involving exogenous TDP-43 fragments. **(A)** Graphic representation of TDP-43 metabolism in cells expressing a *TARDBP* transgene that lacks a 3'-UTR. **(B)** Changes in the level of each element over time when exogenous *TARDBP* mRNA was expressed at a level equivalent to the initial level of the endogenous *TARDBP* mRNA. **(C)** The relative amounts of endogenous TDP-43 in the nucleus (vertical axis) and aggregates (area of circle) are shown when exogenous *TARDBP* expression was increased in a stepwise manner (horizontal axis). **(D)** The relative amounts of endogenous nuclear TDP-43 (vertical axis) and aggregates (area of the circle) compared with the relative amount of extracellular fragmented TDP-43 (horizontal axis) are shown.

We subsequently examined a model in which the *TARDBP* mRNA was exogenously expressed. In most previous experiments, an exogenous *TARDBP* mRNA lacking the 3′-UTR, which is important for autoregulation, was employed. Therefore, this exogenous *TARDBP* mRNA is not autoregulated (Figure 4A, Supplementary Tables 5–7).

First, fluctuations in each factor were examined when the exogenous mRNA was expressed at a level equivalent to the initial level of the endogenous mRNA (Figure 4B). Upon the expression of exogenous TDP-43, the level of the endogenous mRNA was decreased by the autoregulatory mechanism, which was followed by a decrease in endogenous TDP-43 until the level ultimately stabilized at 39% of the initial value (Figure 4B). The total amount of exogenous and endogenous nuclear TDP-43 was 138% of the initial amount of endogenous TDP-43 in the nucleus, which subsequently stabilized. These dynamics are consistent with the results of various previous experiments (Arnold et al., 2013; Koyama et al., 2016).

Next, we changed the expression level of exogenous TDP-43 and investigated the levels of endogenous TDP-43 and its aggregates (Figure 4C). As the level of exogenous TDP-43 increased, the level of endogenous TDP-43 in the nucleus decreased and more TDP-43 aggregates formed. These results are consistent with the results of previous studies in which the overexpression of exogenous TDP-43 reduced endogenous TDP-43 levels and caused aggregates to form in both cell lines and animal models (Xu et al., 2010; Swarup et al., 2011a; McGoldrick et al., 2013).

### Intercellular propagation model of aggregated and fragmented TDP-43

TDP-43 fragmentation is a biochemical property of ALS/FTD, conditions in which TDP-43 is more prone to aggregate, and endogenous TDP-43 may be sequestered with fragmented TDP-43 to form aggregates in affected individuals (Chen et al., 2010; Furukawa et al., 2011). Based on the results of several previous studies using cells cultured in medium supplemented with fragmented TDP-43, the fragmented TDP-43 is transferred into the cells where it forms aggregates with endogenous TDP-43 (Nonaka et al., 2013; Ding et al., 2015; Feiler et al., 2015; Iguchi et al., 2016). We subsequently evaluated this phenomenon using our model. In this model, fragmented TDP-43 was placed outside of the cells, and we set the parameters of the model to ensure that a certain amount of fragmented TDP-43 would be transferred into the cells (Supplementary Table 8). Under these conditions, aggregate formation was promoted when the amount of fragmented TDP-43 reached a certain level, and endogenous intranuclear TDP-43 levels were decreased, similar to the findings in cells from patients affected by ALS/FTD (Figure 4D).

### The half-life of TDP-43

The half-life of total TDP-43 analyzed by stopping translation was 110 relative time units in this model (Supplementary Figure 1). In previous experiments, estimates of the half-life of TDP-43 in cultured cells ranged from 4 to over 34 h, depending on the experimental conditions (Ling et al., 2010; Pesiridis et al., 2011; Watanabe et al., 2012; Scotter et al., 2014). Thus, in our model, the value of 100 relative time units corresponds to a range from 3.6 to over 31 h. Since evidence is not available to determine the half-life of TDP-43 in motor neurons *in vivo*, relative units were also used in the subsequent analysis.

## Analysis of the *in silico* model

### Robustness and fragility of TDP-43 autoregulation

We subsequently examined the fragility of this autoregulatory mechanism by investigating how each element behaves when each parameter was varied. For this analysis, we compared a model with autoregulation [negative autoregulation, NAR (+)] to a model without autoregulation [NAR (–)]. In the NAR (–) model, only the level of transcription was adjusted, which indicates that the initial expression level of the *TARDBP* mRNA was the same as that in the NAR (+) model. Therefore, in a state in which no disturbance was applied, the levels of the *TARDBP* mRNA, nuclear TDP-43, cytoplasmic TDP-43, aggregates, and fragments were equal between these two models. We recorded the amount of each element that stabilized when each parameter was varied from half to twice its initial level.

**Figure 5:**
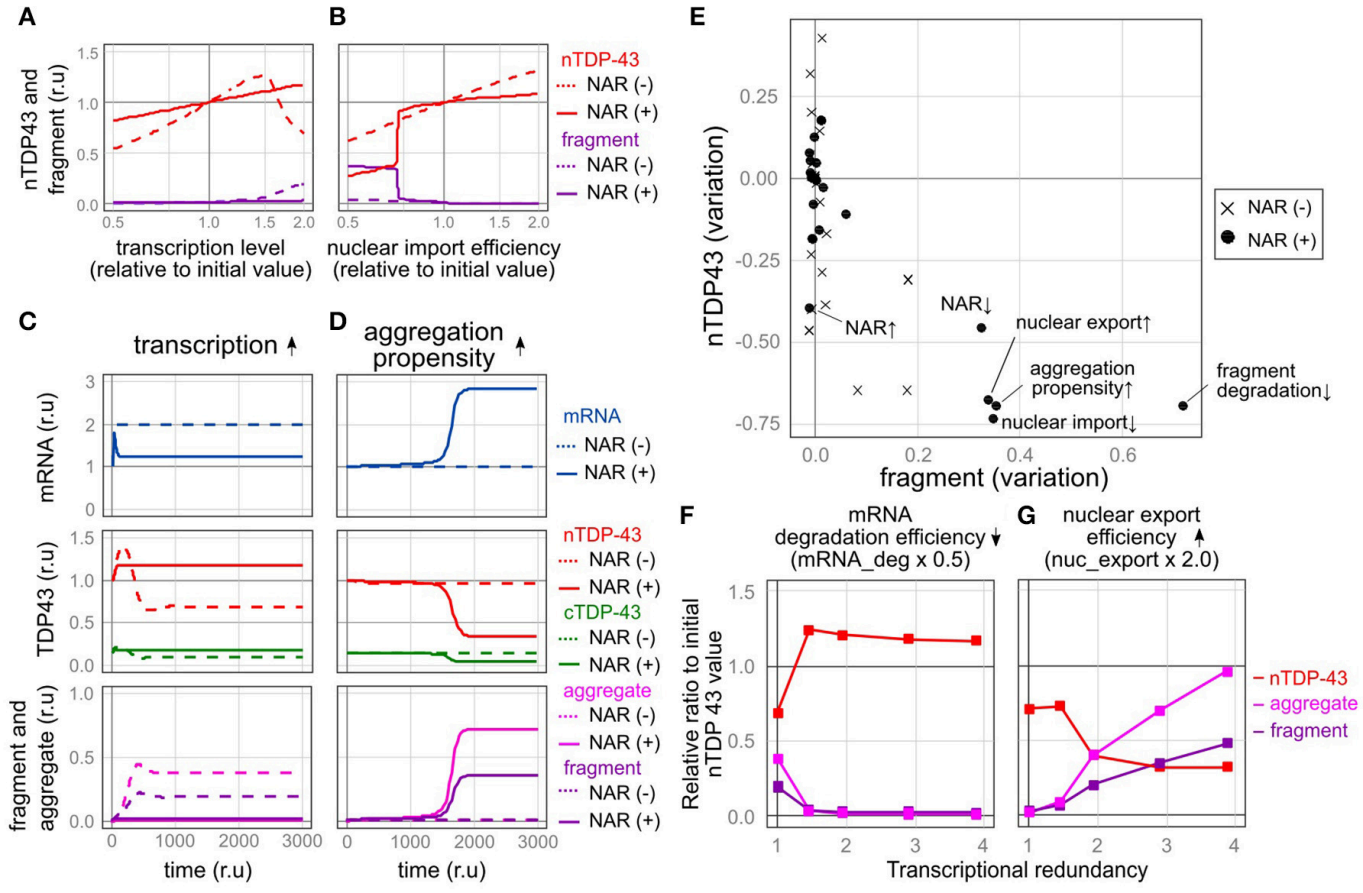
Robustness and fragility of the TDP-43 autoregulatory mechanism. **(A,B)** Plot of the relative amounts of nuclear TDP-43 (red line) and fragments (purple line) that finally stabilized when each parameter was varied (**A**, transcription level; **B**, nuclear import rate) by 100 steps from half to twice the initial value. The dotted line shows the NAR (–) model, and the solid line shows the NAR (+) model. **(C,D)** Changes in the level of each element over time when TDP-43 transcription was increased 2-fold **(C)** or aggregate formation was increased (the parameter agg_K was 0.6 times the initial value) **(D)**. **(E)** The extent of changes in the initial levels of nuclear TPP-43 (vertical axis) and fragmented TDP-43 (horizontal axis) when each parameter was changed to half or twice the initial level is shown. The change in each parameter is indicated by a black circle [NAR (+)] or a cross [NAR (–)]. **(F,G)** Transcriptional redundancy depends on changes in the levels of nuclear TDP-43, aggregates and fragments when each parameter is changed to half or twice the initial level. Representative plots from Supplementary Figure 4 are shown.

The autoregulatory mechanism that controls TDP-43 robustly maintains the nuclear TDP-43 level, even when the levels of many factors were altered. For example, in the NAR (–) model, the level of nuclear TDP-43 decreased as its transcription level decreased, reaching approximately half of the initial value under conditions that mimic the *TARDBP* heterozygous knockout mouse model (Figure 5A). When the transcriptional level increased, the production of TDP-43 increased, and the nuclear TDP-43 level temporarily increased (Figure 5C). The level of cytoplasmic TDP-43 also subsequently increased, resulting in an increase in the levels of fragmented or aggregated TDP-43 accompanied by a decrease in nuclear TDP-43 levels (Figure 5C). However, in the NAR (+) model, regardless of whether transcription was decreased or increased, the nuclear TDP-43 level remained relatively stable (Figures 5A,C). Similar results were obtained when levels of other factors changed (e.g., RNA degradation efficiency, translation efficiency, and TDP-43 degradation efficiency) (Figure 5E and Supplementary Figures 2A, 3A). Thus, the autoregulatory mechanism that maintains the nuclear TDP-43 content is robust.

In contrast, when some factors were changed, the autoregulatory mechanism caused an extreme decrease in the amount of nuclear TDP-43 and an increase in TDP-43 fragmentation, indicating that the autoregulatory mechanism is vulnerable to these factors. For example, the mechanism was vulnerable to a reduction in the nuclear import of TDP-43. In the NAR (−) model, changes in the degree of nuclear import altered the nuclear TDP-43 level; however, these changes did not increase the fragmentation of TDP-43. In the NAR (+) model, the amount of nuclear TDP-43 remained robust until nuclear import was reduced to approximately three-quarters of the initial setting (Figure 5B). When nuclear import fell below this level, the amount of nuclear TDP-43 substantially decreased, whereas the level of fragmented TDP-43 increased (Figure 5B). The mechanism was also vulnerable to an increase in the tendency of TDP-43 to aggregate. In this scenario, a slight but sustained increase in levels of aggregated and fragmented TDP-43 was initially observed; however, the amount of nuclear TDP-43 was strictly maintained for a certain period. At a certain point, the nuclear TDP-43 levels rapidly decreased in parallel with marked increases in the amounts of aggregated TDP-43 and the *TARDBP* mRNA (Figure 5D). In the NAR (–) model, the amount of nuclear TDP-43 remained constant, and no clear increase in levels of aggregation/fragmentation products was identified under the same TDP-43 aggregation-prone conditions (Figure 5D). As the aggregation tendency increases, the nuclear TDP-43 level decreases and nuclear TDP-43 aggregate/fragment accumulation increases in the NAR (–) model; however, the degree of aggregate/fragment accumulation is limited (Supplementary Figure 2B). Similarly, the NAR (+) model exhibited more fragility than the NAR (–) model when the parameters were set such that the efficiency of the degradation of fragmented proteins decreased (Figure 5E and Supplementary Figures 2B, 3B).

### A vicious cycle induced by transcriptional redundancy accelerates TDP-43 pathology

The model in the present study, which was designed to accumulate aggregates depending on the amount of TDP-43 in the cytoplasm, displays bi-stability between healthy and pathological states, depending on the intracellular disturbance. The autoregulatory mechanism, which originally functions to robustly maintain the amount of TDP-43, promotes separation into these two states as the driving force underlying the shift toward TDP-43 pathology when disturbances in specific factors reach or exceed a certain level. Under these conditions, the autoregulatory mechanism is expected to increase the expression of the canonical *TARDBP* mRNA and subsequently exacerbate TDP-43-related pathology (Figure 5D). Indeed, higher expression of the canonical *TARDBP* mRNA is detected in the cytoplasm in the motor neurons from patients with ALS, specifically in motor neurons in which nuclear TDP-43 has been eliminated and inclusion bodies have formed in the cytoplasm (Koyama et al., 2016).

### Trade-off between the robustness and fragility of nuclear TDP-43 homeostasis

We subsequently altered the transcriptional redundancy in five stages to investigate how transcriptional redundancy is involved in TDP-43 metabolism (Supplementary Table 9). We halved or doubled the parameters and subsequently examined the resulting changes in the nuclear TDP-43 levels and the accumulation of aggregates and fragments at each stage (Figures 5F,G and Supplementary Figure 4).

The observed perturbations divided the factors into two types (Figures 5F,G, Supplementary Figures 4A,B). Alterations in one type of factor stabilized the nuclear TDP-43 level as transcriptional redundancy increased (Figure 5F and Supplementary Figure 4A), whereas alterations in the other type of factor led to a marked change in nuclear TDP-43 levels and an increase in TDP-43 aggregates as transcriptional redundancy increased (Figure 5G and Supplementary Figure 4B). The latter type of factors that increased system fragility were the promotion of TDP-43 aggregation, impaired nuclear-cytoplasmic TDP-43 transport, decreased the efficiency of abnormal protein degradation, and reduced efficiency of autoregulation. Thus, the robust autoregulatory mechanism that controls nuclear TDP-43 levels, which is based on transcriptional redundancy, is susceptible to specific factors, potentially because this mechanism is so robust. Therefore, a trade-off between the robustness and fragility of this relationship exists.

### The pathogenesis of hereditary ALS is caused by the fragility of the TDP-43 autoregulatory mechanism

The fragility of the TDP-43 autoregulatory mechanism is manifested by the tendency of TDP-43 to aggregate, impairments in nuclear-cytoplasmic TDP-43 transport, a decrease in the efficiency of the degradation of fragments, and a reduction in autoregulatory efficiency (Figures 5E). Therefore, TDP-43 pathology is triggered if a disorder in one of these factors persistently exceeds a threshold. We therefore suggest that mutations that cause this condition may also cause ALS/FTD.

The causative genes that underlie familial ALS/FTD are intimately associated with these factors. Mutations in the C-terminal low complex region of TDP-43 increase its aggregation-prone nature (Johnson et al., 2009). In cells with an expansion of the GGGGCC repeat at *C9ORF72*, nuclear-cytoplasmic transport is inhibited (Freibaum et al., 2015; Zhang et al., 2015; Boeynaems et al., 2016). Moreover, mutations in *VCP, UBQLNl, SQSTMl, GRN*, and *TBKl* are thought to result in a failure of the degradation machinery, leading to the accumulation of TDP-43 in the cytoplasm (Taylor et al., 2016; Chang et al., 2017; Ramesh and Pandey, 2017).

### TDP-43 pathology may be treated by reductions in transcriptional redundancy

In the present study, we propose that transcriptional redundancy, which is important for the strict regulation of TDP-43, is the driving force underlying the formation and progression of TDP-43 pathology in patients with ALS. We therefore hypothesize that strategies that reduce transcriptional redundancy may affect TDP-43 pathology in patients with ALS. We evaluated this possibility using the model developed by our group described below.

**Figure 6:**
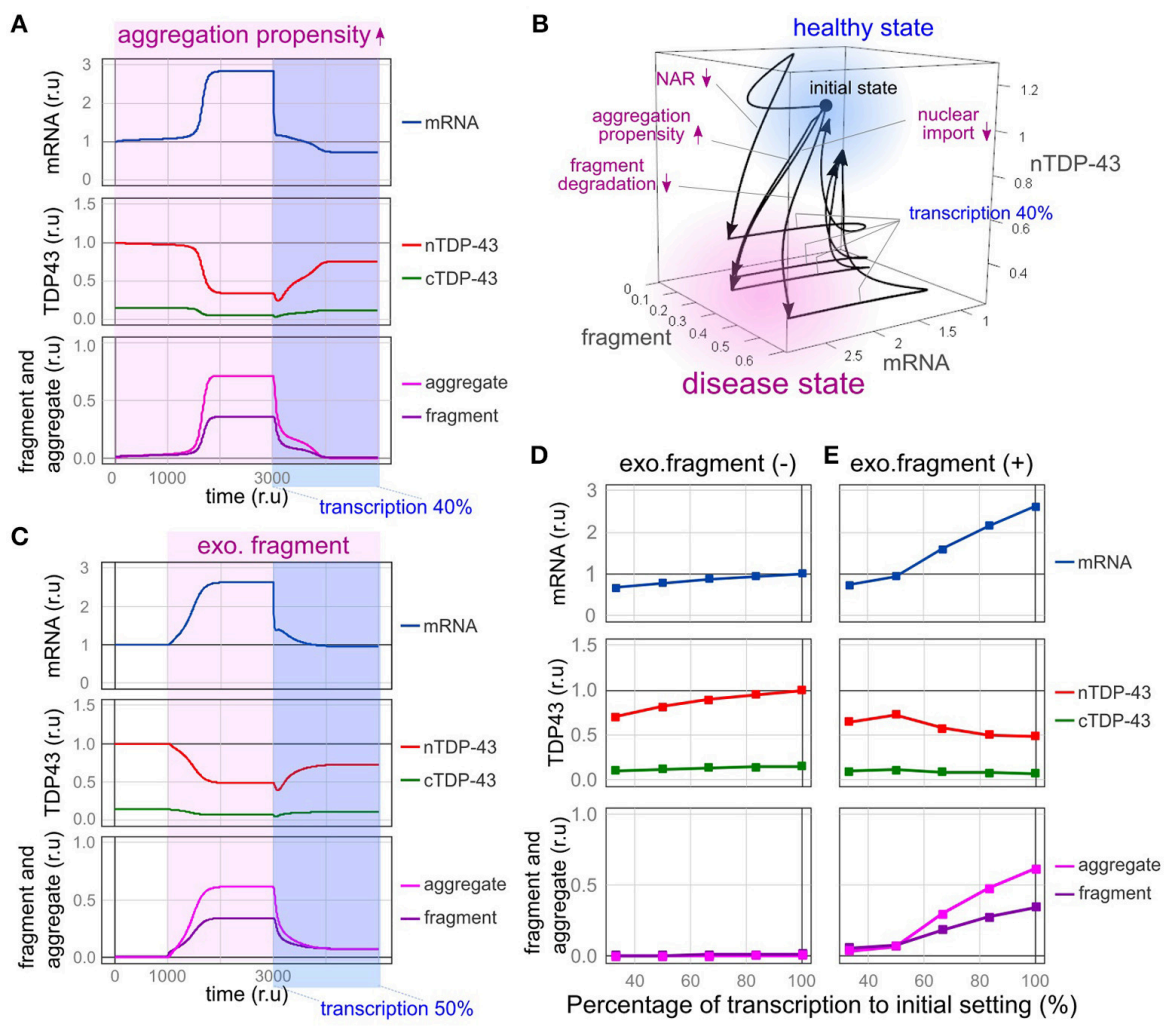
Therapeutic effects of reducing the transcriptional redundancy of TDP-43. **(A)** Changes in the level of each element over time are shown when transcription is reduced to 40% in a state in which TDP-43 tends to aggregate. **(B)** Due to the disturbance shown in the figure, the system enters a disease state (pale red region) exhibiting an increase in mRNA levels, a decrease in nuclear TDP-43 levels, and an increase in fragment levels. When transcription is reduced to 40%, the system returns to a healthy state (light blue region). **(C)** Changes in the level of each element over time are shown when transcription is reduced to 50% in a state in which the amount of extracellular fragments is increased. **(D,E)** Changes in each element related to the decrease in transcription are shown under the condition in which the same amount of extracellular fragments depicted in **C** is present **(E)** or absent **(D)**.

First, we modeled the conditions under which the formation of TDP-43 aggregates is continuously increased by the mutation of *TARDBP* and the formation of stress granules is promoted. *TARDBP* mRNA levels increased, nuclear TDP-43 levels decreased, and aggregated/fragmented TDP-43 accumulation increased over time, as shown in Figure 5D. However, after the transcription of the *TARDBP* mRNA decreased to 40%, nuclear TDP-43 levels increased, fragmented TDP-43 levels decreased and subsequently disappeared, and the *TARDBP* mRNA level returned to a nearly normal value (Figure 6A). Based on these results, TDP-43 pathology may be rescued by reducing the redundancy of *TARDBP* transcription.

Impaired nuclear-cytoplasmic TDP-43 transport, decreased efficiency of abnormal protein degradation, and reduced autoregulatory efficiency also shift the system to a disease state [Figure 6B, from a healthy state (light blue) to a disease state (pale red)]. However, the bi-stability of healthy and disease states stratified by the absence or presence of these disturbances became ambiguous when transcriptional redundancy was reduced. Therefore, disease conditions induced by these disturbances were improved (Figure 6B, from a disease state (pale red) to a healthy state (light blue); and Supplementary Figure 5). Although the factors that cause sporadic ALS in humans remain obscure, strategies that reduce transcriptional redundancy may represent a treatment option for ALS in patients with TDP-43 pathology initiated by any of the factors implicated in this model.

### Reduced transcription inhibits the propagation of TDP-43 pathology

We speculated that a reduced transcription level would reduce the formation of new TDP-43 aggregates and fragments and subsequently suppress the propagation of pathological TDP-43 to other neurons. We therefore evaluated this hypothesis using the model developed by our group.

In this model, fragmented TDP-43 was assumed to be continuously propagated to other neurons to a certain extent, regardless of its propagation pattern. As shown in Figure 4D, TDP-43 pathology subsequently developed in the receiving neuron. When the transcription of TDP-43 was reduced to half in the receiving neuron, nuclear TDP-43 levels increased, the numbers of TDP-43 aggregates and fragments decreased, and the mRNA level recovered to near-normal values in the receiving neuron (Figure 6C). Thus, the spread of TDP-43 lesions mediated by fragmented TDP-43 was also rescued by decreasing its transcription.

We predicted that the spread of this pathological state would be affected by the amount of extracellular fragmented TDP-43. Therefore, we examined the extent of extracellular fragmentation of TDP-43 at six concentrations and determined the extent of the spread of the pathological state to receiving cells (Figures 6D,E and Supplementary Figure 6). When relatively small amounts of extracellular TDP-43 fragments were present, the receiving cell did not exhibit a pathological state (Supplementary Figures 6A–C). However, when the amount of extracellular TDP-43 fragments exceeded a certain level, the nuclear TDP-43 level decreased, and TDP-43 aggregates accumulated in the cytoplasm of the receiving cell (Supplementary Figures 6D–F). However, depending on the extent of the reduction in transcription in the receiving cell, the transition to this pathological condition was impeded (Figure 6E and Supplementary Figure 6). Thus, a therapeutic strategy designed to reduce the transcriptional redundancy of TDP-43 would prevent not only the accumulation and supply of aggregates and fragments of TDP-43 but also the spread of the disease state between cells.

## Discussion

**Figure 7:**
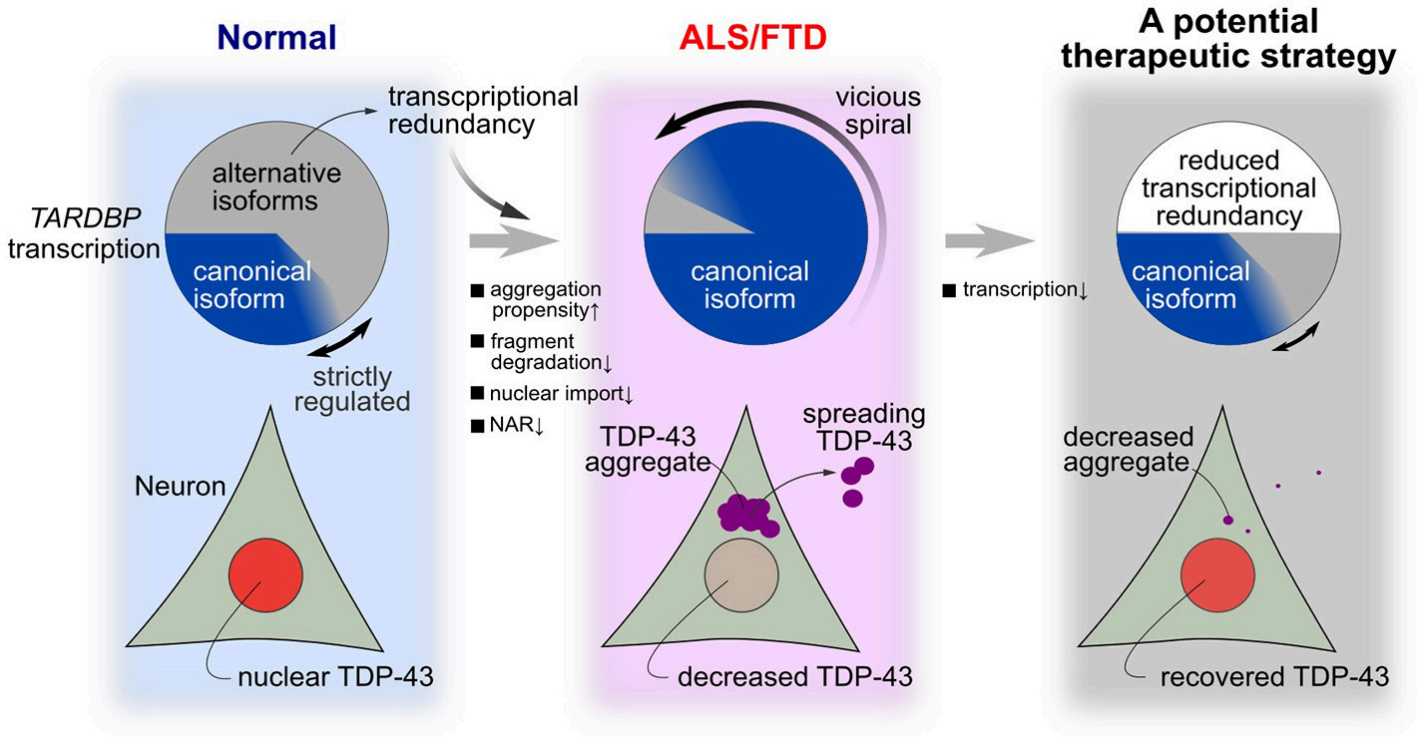
A hypothesis for the mechanism of TDP-43 pathology in ALS and a potential treatment strategy. In a normal state, many transcribed RNAs are ultimately rapidly degraded via nonsense-mediated mRNA decay. Strict autoregulation is maintained by changing the ratios of alternatively spliced isoforms according to the amount of TDP-43 present in the nucleus. However, this robust mechanism for maintaining nuclear TDP-43 levels becomes fragile when certain intracellular lesions persist at a constant intensity. In these cases, the level of nuclear TDP-43 substantially decreases, aggregation/fragmentation increases, and the level of canonical *TARDBP* mRNA increases until the model enters the pathological state of ALS/FTD. Transcriptional redundancy promotes the transition to this pathological condition. A therapeutic strategy that reduces transcription by approximately one-half abolishes transcriptional redundancy and may reverse the morbidity observed in patients with ALS.

In the present study, we attempted to identify specific factors related to the fragility of the TDP-43 autoregulatory mechanism using an *in silico* model mimicking the intracellular dynamics of TDP-43. TDP-43 autoregulation involves transcriptional redundancy and is robust to disturbances in many factors (Figure 7, left panel). However, when specific factors exceed a certain threshold for a certain period, the fragility of the autoregulatory mechanism becomes apparent. This robust autoregulatory mechanism based on transcriptional redundancy shows vulnerability under specific pathological conditions and is prone to falling into a vicious cycle in which the TDP-43 pathology continuously accelerates and deteriorates (Figure 7, center panel). Therefore, the system controlling TDP-43 levels shows bi-stability between healthy and disease states, according to the circumstances, and these properties of robustness and fragility may underlie the pathogenesis of TDP-43 pathology.

An appropriate amount of nuclear TDP-43 must be constantly maintained to establish homeostasis of RNA metabolism. In motor neurons, the intracellular environment may be disturbed by various factors; thus, motor neurons likely require a system that is robust to these disturbing factors. The model of cellular TDP-43 metabolism proposed here robustly maintains the amount of TDP-43 in the nucleus at a constant level in the presence of several types of disturbances, including disturbances in transcription, RNA degradation, translation, and the efficiency of degradation of the TDP-43 protein. In addition, the system was stable when fluctuations in nuclear-cytoplasmic TDP-43 transport and the increase in the tendency of the protein to aggregate were mild or persisted for a short time. However, when these disturbances were intense and sufficiently prolonged, the vulnerability of the autoregulatory system became apparent. According to several studies, TDP-43 expression increases under stress conditions, such as neuronal injury (Moisse et al., 2009), or in response to neural activity (Wang et al., 2008); however, the mechanism underlying this context-dependent change in TDP-43 expression has not been elucidated. Due to the existence of the TDP-43 autoregulatory mechanism, fluctuations in its transcription, translation efficiency, and protein degradation efficiency are less likely to affect its expression level. Therefore, in these cell states, a decrease in the efficiency of the autoregulatory mechanism itself may increase TDP-43 expression. Further studies are required to investigate whether transcriptional redundancy also contributes to the rapid increase in expression required depending on the situation within the cell. Based on the results obtained from the *in silico* model, the system will eventually shift to a disease state as the efficiency of the autoregulatory mechanism decreases and transcriptional redundancy persists. The relationship between environmental factors in motor neurons and the efficiency of the TDP-43 autoregulatory mechanism should be investigated.

At this stage, we propose that TDP-43 pathology involves a vicious cycle in which excessive TDP-43 is continuously produced as a result of an autoregulatory mechanism. As shown in a previous study, motor neurons from subjects with ALS displaying abnormal TDP-43 accumulation exhibit an increase in the amount of *TARDBP* mRNA in the cytoplasm (Koyama et al., 2016), whereas other studies have observed higher amounts of TDP-43 in the cerebrospinal fluid and brain-derived exosomes obtained from patients with ALS (Kasai et al., 2009; Iguchi et al., 2016). The genetic background that results in impaired control of TDP-43 levels has not been sufficiently investigated. A family with ALS presenting with a mutation in the *TARDBP* 3′-UTR that leads to an increase in *TARDBP* mRNA levels has been identified. This mutation is located near the splice site of intron 7, which plays an important role in autoregulation, and it would be interesting to determine whether the mechanism that causes this increase involves the autoregulatory capacity of this gene (Gitcho et al., 2009). Interestingly, mutations in the *TARDBP* gene associated with ALS are frequently identified in intron 6 (Onodera et al., 2013), which undergoes alternative splicing and plays an important role in autoregulation (Koyama et al., 2016). These mutations may result in changes in the sequence motif to which the splicing factor binds, potentially causing autoregulatory abnormalities resulting from changes in the splicing efficiency or aberrations in cis factors. This possibility should be explored in the future. Furthermore, the genes that have been identified to date as causing familial ALS with TDP-43 pathology (*TARDBP, C9ORF72, VCP, UBQLNl, SQSTMl, GRN*, and *TBKl*) are related to disturbances in the factors that lead to the vicious cycle of the autoregulatory system. Therefore, examinations of the TDP-43 metabolism and specifically the expression of *TARDBP* mRNA in model animals with mutations in these causative genes are necessary.

Using this model, TDP-43 pathology was reversed and abnormal TDP-43 propagation was suppressed when the transcription of the *TARDBP* mRNA was suppressed (Figure 7, right panel). Mice conditionally overexpressing the TDP-43 mutant that lacked a nuclear localization signal showed TDP-43 pathology and neurological symptoms; however, when protein expression was terminated, the TDP-43 pathology was reversed and the loss of neurological function was abolished (Walker et al., 2015). Similar results were reported in mice that conditionally overexpressed TDP-43 containing an ALS-related mutation (Ke et al., 2015). Based on these findings, strategies alleviating TDP-43 overexpression ameliorate the disease state. We should validate this therapeutic strategy of reducing *TARDBP* mRNA levels under the pathological condition of persistent *TARDBP* mRNA expression driven by transcriptional redundancy. An animal model that maintains the autoregulatory mechanism that controls TDP-43 levels is necessary to provide supporting evidence for this treatment strategy. In this type of animal model, the endogenous *TARDBP* mRNA, but not exogenous *TARDBP*, should be overexpressed, as observed in motor neurons from patients with ALS (Koyama et al., 2016). This animal model should ideally be accompanied by a perturbation that induces the fragility of the TDP-43 autoregulatory system due to mutations in ALS causative genes. Once this ALS animal model is established, we can test the therapeutic effectiveness of strategies designed to reduce the transcriptional redundancy of *TARDBP* mRNA in ALS. Specifically, a strategy that employs an antisense oligonucleotide or CRISPR-dCas9 system to decrease the *TARDBP* mRNA level would conceivably be successful. A decrease in TDP-43 levels induces a loss in TDP-43 function, which may be detrimental to cell survival and function. However, because *TARDBP* expression is transcriptionally redundant, a 50% reduction in its transcription does not affect protein levels (Kraemer et al., 2010; Sephton et al., 2010; Ricketts et al., 2014). Moreover, *TARDBP* heterozygous knockout mice have normal survival times (Ricketts et al., 2014). Therefore, treatments that target *TARDBP* mRNA levels might be effective.

The pathophysiology of neurodegenerative diseases is particularly difficult to understand due to the complexity, multifactorial, and dynamic aspects of the central nervous system. Approaches using systems biology may be useful for integrating all aspects of a given phenomenon and may constitute promising strategies. However, models of any scale have advantages and limitations (Vujasinovic et al., 2010). We have created a small-scale, simple model that focuses on the dynamics of TDP-43 and does not contain other related molecules. In reality, however, intracellular metabolism, including TDP-43 metabolism, may occur while many unidentified molecules interact. Extracellular factors, including the mechanism of TDP-43 propagation between cells, are also poorly understood. Therefore, we cannot accurately model all of these factors at this stage. In addition, previous experimental results on the temporal aspects of each element, such as the half-life of the wild-type TDP-43 protein, vary greatly, depending on the cell type and conditions used in the experiment. We thus adopted relative times because we were concerned that descriptions of models incorporating absolute times would lead to erroneous interpretations. A decrease in the abstraction level or an increase the scale of the model by increasing the number of factors in the model increases the difficulty of setting appropriate parameters in the model and may result in a misinterpretation of the results. Meanwhile, even if many other factors are involved, we rationally presume that these factors ultimately act on the dynamics of TDP-43 and are responsible for the development of ALS-related TDP-43 pathology. Therefore, we postulate that the scale and abstraction level of this model are suitable for the research purpose to reveal hidden vulnerabilities in the robustness of the TDP-43 autoregulatory mechanism. However, changes in the intracellular environment may influence multiple factors that determine the dynamics of TDP-43. In addition, since secondary impairments in nuclear-cytoplasmic TDP-43 transport due to the accumulation of aggregates have been reported (Woerner et al., 2015), disturbances in multiple factors may occur continuously. Despite this evidence, the present study did not analyze pathological conditions involving these multiple factors. The hypothesis and treatment strategy for TDP-43 pathology proposed based on this model should be experimentally verified as discussed above. In addition, the relationship of each element and the scale of the model can be corrected based on accumulating results from biological experiments.

## Conclusion

Why does TDP-43 accumulate abnormally in the cytoplasm in patients with ALS/FTD when a mechanism that precisely maintains its expression exists? Studies aiming to resolve this issue are important to enhance our understanding of the pathology of sporadic ALS, which accounts for most ALS cases, and to promote the development of methods to treat this disease. Therefore, in the present study, we modeled and simulated TDP-43 dynamics within the cell. The trade-off relationship between the vulnerability and robustness of the mechanism that maintains nuclear TDP-43 levels might underlie TDP-43 pathology. Once this vulnerability is triggered, the redundancy of *TARDBP* transcription that is intended to maintain nuclear TDP-43 levels becomes the driving force that leads to TDP-43 pathology. This hypothesis answers several questions: why do ALS motor neurons enter a pathological state from which they cannot escape; why do many different ALS causative genes result in TDP-43 pathology; and why does TDP-43 pathology propagate between cells, thereby resulting in progression? Furthermore, based on our model, therapies for ALS targeting specific molecules are a possibility. In the future, an ideal animal model of this disease should be established to explore this hypothesis.

## Author contributions

AS and OO designed the study. AS, TKA, and AK performed the biological experiments using the mouse cerebrum and investigated the results of previous experiments to create our model. AS analyzed the results. YK, SK, TKO, and TI contributed to the interpretation of the findings. AS and OO drafted the manuscript. All authors critically revised the draft and approved the final version.

### Conflict of interest statement

The authors declare that the research was conducted in the absence of any commercial or financial relationships that could be construed as a potential conflict of interest.
