# Supplementary material for: Robustness and Vulnerability of the Autoregulatory System That Maintains Nuclear TDP-43 Levels: A Trade-off Hypothesis for ALS Pathology Based on in Silico Data
Source: Front Neurosci. 2018 Feb 1;12:28. doi: 10.3389/fnins.2018.00028 (PMC5799296; doi:10.3389/fnins.2018.00028)
Supplement: Supplementary file 1 [file Presentation1.pdf]

Supplementary Material

Robustness and vulnerability of the autoregulatory system that maintains nuclear TDP-43 levels: a trade-off hypothesis for ALS pathology based on *in silico* data

Akihiro Sugai[1*], Taisuke Kato[2], Akihide Koyama[3], Yuka Koike[1], Sou Kasahara[1], Takuya Konno[1], Tomohiko Ishihara[4], Osamu Onodera[1*]

[1]Department of Neurology, Clinical Neuroscience Branch, Brain Research Institute, Niigata University, Niigata, Japan

[2]Department of System Pathology for Neurological Disorders, Brain Science Branch, Center for Bioresource-based Research, Brain Research Institute, Niigata University, Niigata, Japan

[3]Division of Legal Medicine, Graduate School of Medicine and Dental Science, Niigata University, Niigata, Japan

[4]Department of Molecular Neuroscience, Resource Branch for Brain Disease Research, Center for Bioresource-based Research, Brain Research Institute, Niigata University, Niigata, Japan

*** Correspondence:**

Akihiro Sugai: akihiro.sugai@bri.niigata-u.ac.jp

Osamu Onodera: onodera@bri.niigata-u.ac.jp

1 Supplementary Tables

Supplementary Table 1. Math of reactions in the model with autoregulation.

Id	Reactants	Products	Modifiers	Math
re1	s2 (pre-mRNA)	s3 (mRNA)	s5 (nTDP43)	s2 * k2/(1 + pow(s5 / Knar, n))
re2	s3 (mRNA)	s8 (nucleic acids)		s3 * k3
re3	s4 (cTDP43)	s5 (nTDP43)		s4 * k7
re4	s4 (cTDP43)	s6 (fragment), s10 (amino acids)		Vfr * s4/(1 + pow(Kfr / s4, n1))
re5	s4 (cTDP43)	s7 (aggregate)	s6 (fragment)	Vag * s4/(1 + pow(Kag/s6, n2))
re6	s4 (cTDP43)	s10 (amino acids)		s4 * k5
re7	s5 (nTDP43)	s4 (cTDP43)		s5 * k8
re8	s5 (nTDP43)	s11 (amino acids)		s5 * k6
re9	s6 (fragment)	s10 (amino acids)		s6 * k9
re10	s7 (aggregate)	s6 (fragment)		s7 * k10
re11	s7 (aggregate)	s10 (amino acids)		s7 * k11
re12	s9 (nucleic acids)	s2 (pre-mRNA)	s1 (*TARDBP*)	s1 * k1
re13	s10 (amino acids)	s4 (cTDP43)	s3 (mRNA)	s3 * k4

Supplementary Table 2. Initial quantities in the model with autoregulation.

Id	Name	Compartment	Initial Quantity	Boundary condition	Constant
s1	*TARDBP*	nucleus	2.00	TRUE	TRUE
s2	pre-mRNA	nucleus	3.00	TRUE	TRUE
s3	mRNA	cytoplasm	10.323	FALSE	FALSE
s4	cTDP43	cytoplasm	176.25	FALSE	FALSE
s5	nTDP43	nucleus	1,174.77	FALSE	FALSE
s6	fragment	cytoplasm	15.35	FALSE	FALSE
s7	aggregate	cytoplasm	3.53	FALSE	FALSE
s8	cyto. nucleic acids	cytoplasm	1.00	TRUE	TRUE
s9	nuc. nucleic acids	nucleus	1.00	TRUE	TRUE
s10	cyto. amino acids	cytoplasm	1.00	TRUE	TRUE
s11	nuc. amino acids	nucleus	1.00	TRUE	TRUE

Supplementary Table 3. Parameters in the models with autoregulation.

Id	Name	Value
k1	transcription	1.00
k2	post_transcription	1.00
Knar	NAR	1,000
n	NAR_hill	4.00
k3	mRNA_deg	0.10
k4	translation	1.00
k5	cTDP43_deg	0.02
k6	nTDP43_deg	0.005
k7	nuc_import	0.10
k8	nuc_export	0.01
Vfr	frag_V	0.60
Kfr	frag_K	2,000
n1	frag_hill	2.00
k9	frag_deg	0.06
Vag	agg_V	0.66
Kag	agg_K	500
n2	agg_hill	2.00
k10	agg_frag	0.03
k11	agg_deg	0.001

Supplementary Table 4. Primers and probes used for droplet digital PCR.

Primer/Probe	Name	Sequcence	Modification
Forward primer	TdpEx3-4 FWD	AACTGAGCAGGATCTGAAAGAC	
Reverse primer	TdpEx3-4 REV	CGAACAAAGCCAAACCCTTTC	
FAM Probe	TdpEx3-4 PRB	TGGAGAGGTTCTTATGGTTCAGGTCA	Int ZEN, 5' 6-FAM, 3' Iowa Black® FQ
Forward primer	Int6 FWD	AGGTGGCTTTGGGAATCAG	
Reverse primer	Int6 REV	CACCAAAGTTCATCCCTCCA	
HEX Probe	Int6 PRB_Hex	TGGAGCTGGCTTGGGAAATAACCA	Int ZEN, 5' HEX, 3' Iowa Black® FQ

Supplementary Table 5. Math of reactions in the model with exogenous *TARDBP* expression.

Id	Reactants	Products	Modifiers	Math
re001	s002 (pre-mRNA)	s003 (mRNA)	s005,s105	s002 * k002 / (1 + pow((s005 + s105) / Knar, n))
re002	s003 (mRNA)	s008 (nucleic acids)		s003 * k003
re003	s004 (cTDP43)	s005 (nTDP43)		s004 * k007
re004	s004 (cTDP43)	s006 (fragment), s010 (amino acids)		Vfr * s004 / (1 + pow(Kfr / s004, n1))
re005	s004 (cTDP43)	s007 (aggregate)	s006 (fragment)	Vag * s004 / (1 + pow(Kag / s006, n2))
re006	s004 (cTDP43)	s010 (amino acids)		s004 * k005
re007	s005 (nTDP43)	s004 (cTDP43)		s005 * k008
re008	s005 (nTDP43)	s011 (amino acids)		s005 * k006
re009	s006 (fragment)	s010 (amino acids)		s006 * k009
re010	s007 (aggregate)	s006 (fragment)		s007 * k010
re011	s007 (aggregate)	s010 (amino acids)		s007 * k011
re012	s009 (nucleic acids)	s002 (pre-mRNA)	s001 (*TARDBP*)	s001 * k001
re013	s010 (amino acids)	s004 (cTDP43)	s003 (mRNA)	s003 * k004
re101	s102 (exo_pre-mRNA)	s103 (exo_mRNA)		s102 * k102
re102	s103 (exo_mRNA)	s008 (nucleic acids)		s103 * k103
re103	s104 (exo_cTDP43)	s105 (exo_nTDP43)		s104 * k107
re104	s104 (exo_cTDP43)	s006 (fragment), s010 (amino acids)		Vm * s104 / (1 + pow(Kfr / s104, n1))
re105	s104 (exo_cTDP43)	s007 (aggregate)	s006 (fragment)	Vag * s104 / (1 + pow(Kag / s006, n2))
re106	s104 (exo_cTDP43)	s010 (amino acids)		s104 * k105
re107	s105 (exo_nTDP43)	s104 (exo_cTDP43)		s105 * k108
re108	s105 (exo_nTDP43)	s011 (amino acids)		s105 * k106
re112	s009 (nucleic acids)	s102 (exo_pre-mRNA)	s101 (exo_*TARDBP*)	s009 * k101
re113	s010 (amino acids)	s104 (exo_cTDP43)	s103 (exo_mRNA)	s103 * k104

Supplementary Table 6. Initial quantities in the model with exogenous *TARDBP* expression.

Id	Name	Compartment	Initial Quantity	Boundary condition	Constant
s001	*TARDBP*	nucleus	2.00	TRUE	TRUE
s002	pre-mRNA	nucleus	3.00	TRUE	TRUE
s003	mRNA	cytoplasm	10.32	FALSE	FALSE
s004	cTDP43	cytoplasm	176.25	FALSE	FALSE
s005	nTDP43	nucleus	1174.77	FALSE	FALSE
s006	fragment	cytoplasm	15.35	FALSE	FALSE
s007	aggregate	cytoplasm	3.53	FALSE	FALSE
s008	cyto. nucleic acids	cytoplasm	1.00	TRUE	TRUE
s009	nuc. nucleic acids	nucleus	1.00	TRUE	TRUE
s010	cyto. amino acids	cytoplasm	1.00	TRUE	TRUE
s011	nuc. amino acids	nucleus	1.00	TRUE	TRUE
s101	exo_*TARDBP*	nucleus	1.00	TRUE	TRUE
s102	exo_pre-mRNA	nucleus	1.03	TRUE	TRUE
s103	exo_mRNA	cytoplasm	0.00	FALSE	FALSE
s104	exo_cTDP43	cytoplasm	0.00	FALSE	FALSE
s105	exo_nTDP43	nucleus	0.00	FALSE	FALSE

Supplementary Table 7. Parameters in the models with exogenous *TARDBP* expression.

Id	Name	Value
k001	transcription	1.00
k002	post transcription	1.00
Knar	NAR	1000
n	NAR_hill	4.00
k003	mRNA_deg	0.10
k004	translation	1.00
k005	cTDP43_deg	0.02
k006	nTDP43_deg	0.01
k007	nuc_import	0.10
k008	nuc_export	0.01
Vfr	frag_V	0.60
Kfr	frag_K	2000
n1	frag_hill	2.00
k009	frag_deg	0.06
Vag	agg_V	0.66
Kag	agg_K	500
n2	agg_hill	2.00
k010	agg_frag	0.03
k011	agg_deg	0.00
k101	exo_transcription	1.00
k102	exo_post_transcription	1.00
k103	exo_mRNA_deg	0.10
k104	exo_translation	1.00
k105	exo_cTDP43_deg	0.02
k106	exo_nTDP43_deg	0.01
k107	exo_nuc_import	0.10
k108	exo_nuc_export	0.01
Vm	frag_V	0.60
Kfr	frag_K	2000
n1	frag_hill	2.00
Vag	agg_V	0.66
Kag	agg_K	500
n2	agg_hill	2.00

Supplementary Table 8. Math of reaction in the models with exogenous TDP-43 fragments.

Id	Reactants	Products	Modifiers	Math
re14	s12 (exo-fragment)	s6 (fragment)		s12 * k12

The other parameters and initial values are the same as those of NAR (+) model. The reactant s12 (exo-fragment) is a constant and its initial quantity in Supplementary Figure 6f is set to 50. The parameter k12 is set to 0.1.

Supplementary Table 9. Parameter setting for different transcriptional redundancy.

transcriptional redundancy	s2 (pre-mRNA)	Knar
1.00	1.0323	-
1.45	1.5000	1,432.00
1.94	2.0000	1,194.00
2.91	3.0000	1,000.00
3.87	4.0000	902.27
4.84	5.0000	839.10

The other parameters and initial values are the same as those of the NAR (+) model (Supplementary Tables 1-3).

2 Supplementary Figures

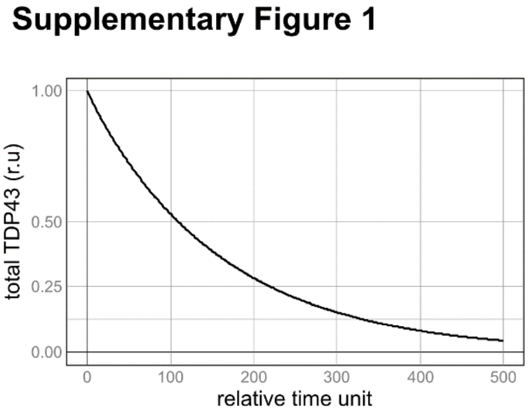

Supplementary Figure 1. The half-life of total TDP-43. Analysis of translation termination showed that the half-life of TDP-43 was 110 relative time units in this model.

Supplementary Figure 2

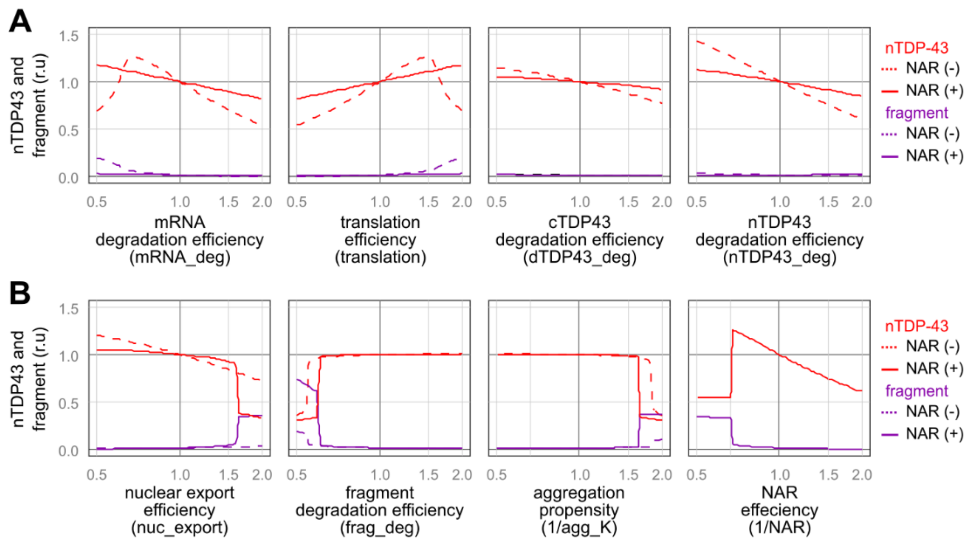

Supplementary Figure 2. Changes in the level of each element according to the degree of each disturbance. **(A, B)** Plot of the relative amounts of nuclear TDP-43 (red line) and fragments (purple line) that finally stabilized when varying each parameter by 100 steps from half to twice the initial set value. The dotted line shows the NAR (-) model, and the solid line shows the NAR (+) model. **(A)** Parameter fluctuation when the NAR (+) model is robust. **(B)** Parameter fluctuation when the NAR (+) model is more vulnerable.

Supplementary Figure 3

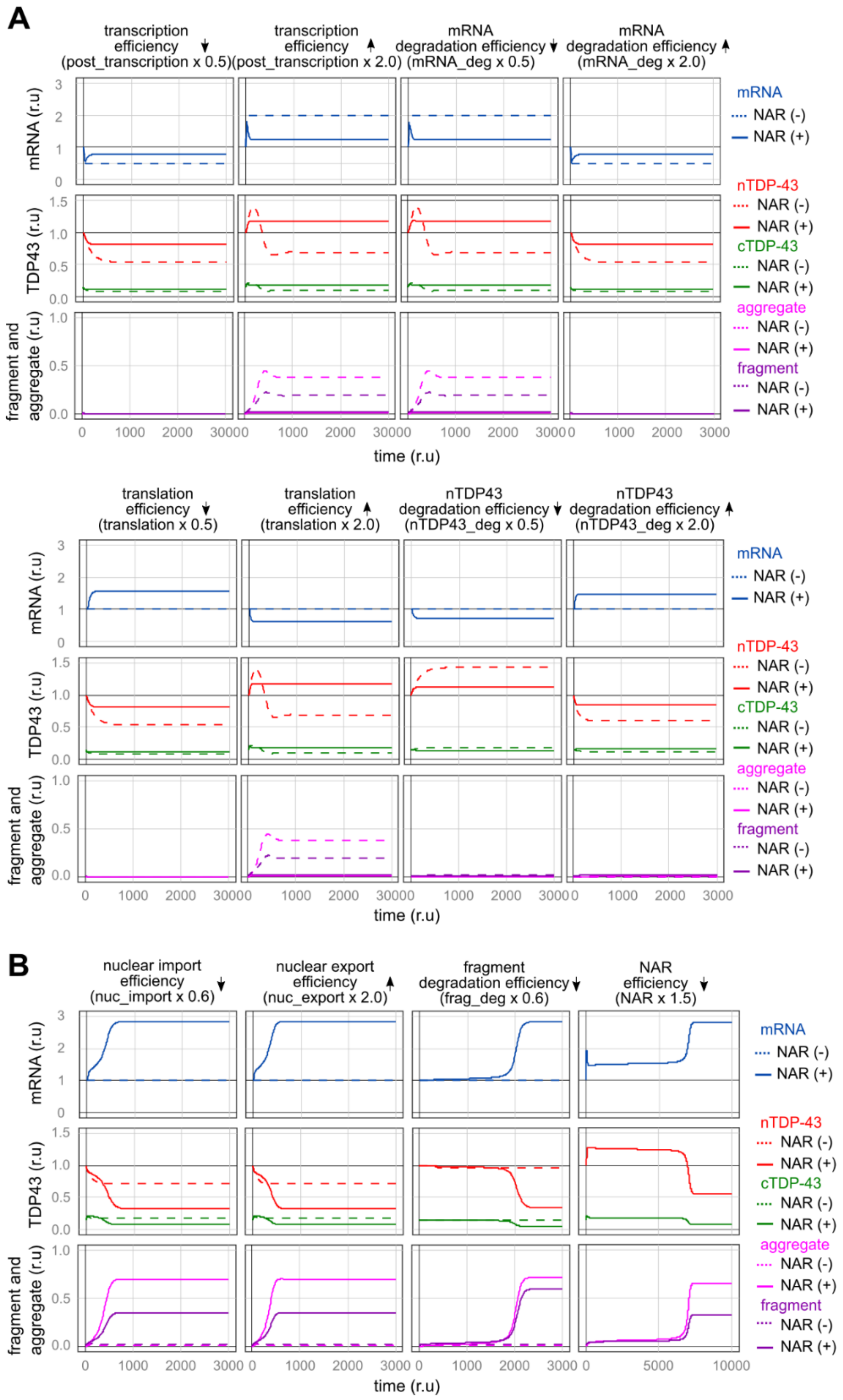

Supplementary Figure 3. Time course of each element according to changes in each parameter. **(A, B)** Changes over time in the level of each element when each parameter is changed as shown. The dotted line shows the NAR (-) model, and the solid line shows the NAR (+) model. **(A)** Parameter changes when the NAR (+) model is robust. **(B)** Parameter changes when the NAR (-) model is more vulnerable.

Supplementary Figure 4

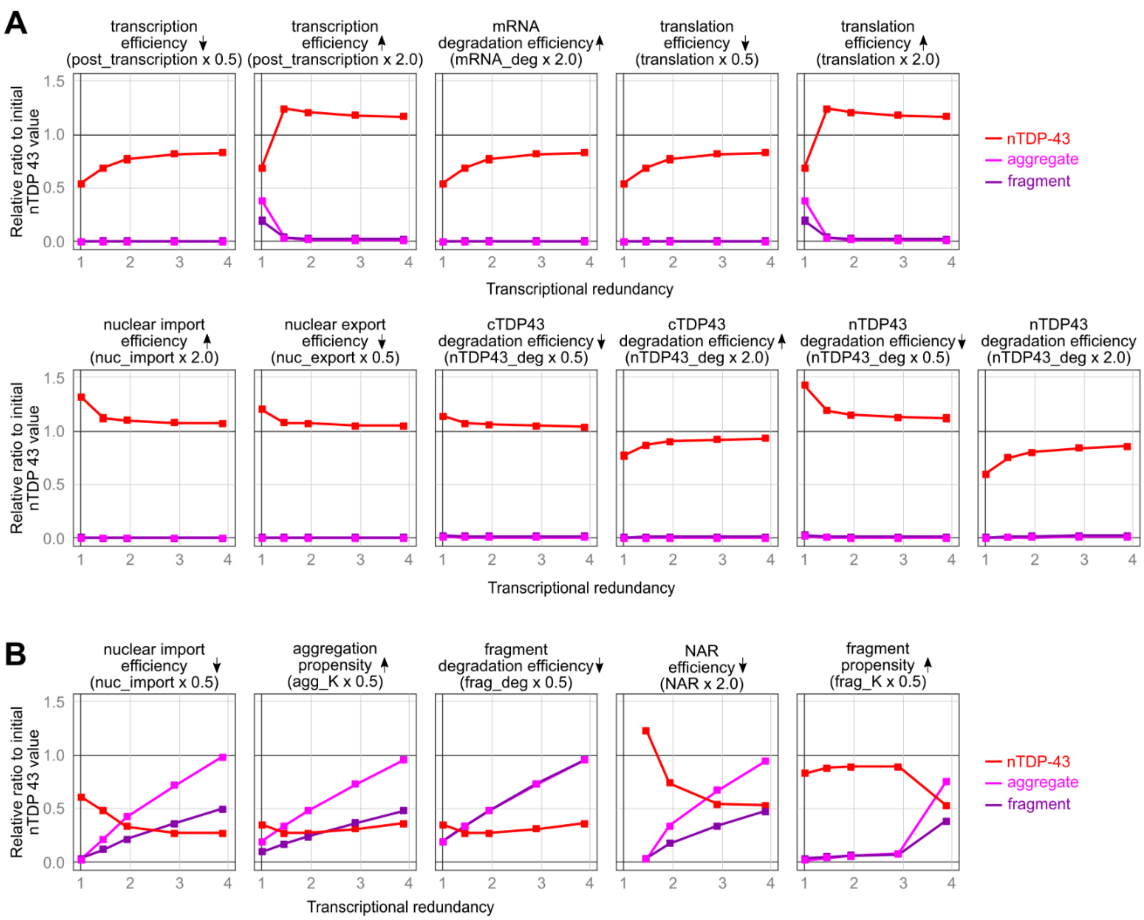

Supplementary Figure 4. Robustness and vulnerability due to transcriptional redundancy (Supplement to Figure 5F-G). **(A, B)** Transcriptional redundancy dependency change in each element when each parameter is changed to half or twice the initial value. **(A)** Parameter changes when the system becomes more robust as transcriptional redundancy increases. **(B)** Parameter changes when the system becomes more vulnerable as transcriptional redundancy increases.

Supplementary Figure 5

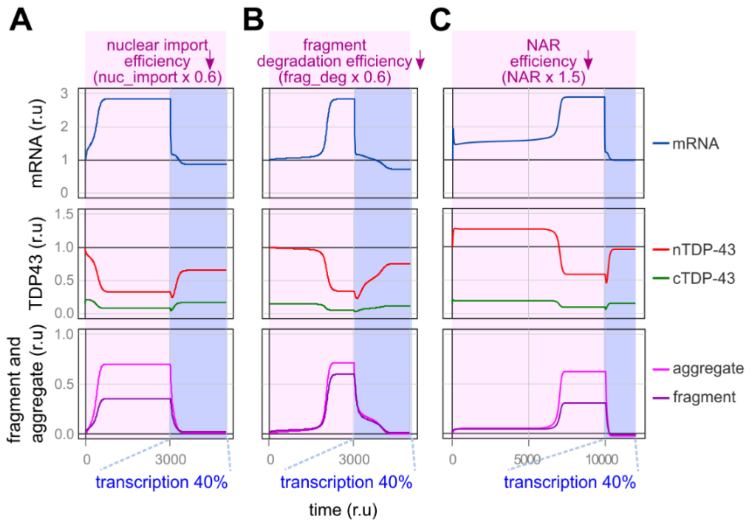

Supplementary Figure 5. Therapeutic effects on TDP-43 pathology (Supplement to Figure 6B). **(A, B, C)** Changes over time in the level of each element are shown when transcription is reduced to 40% in a state in which nuclear import is reduced **(A)**, fragment degradation is decreased **(B)**, or autoregulation efficiency is decreased **(C)**.

Supplementary Figure 6

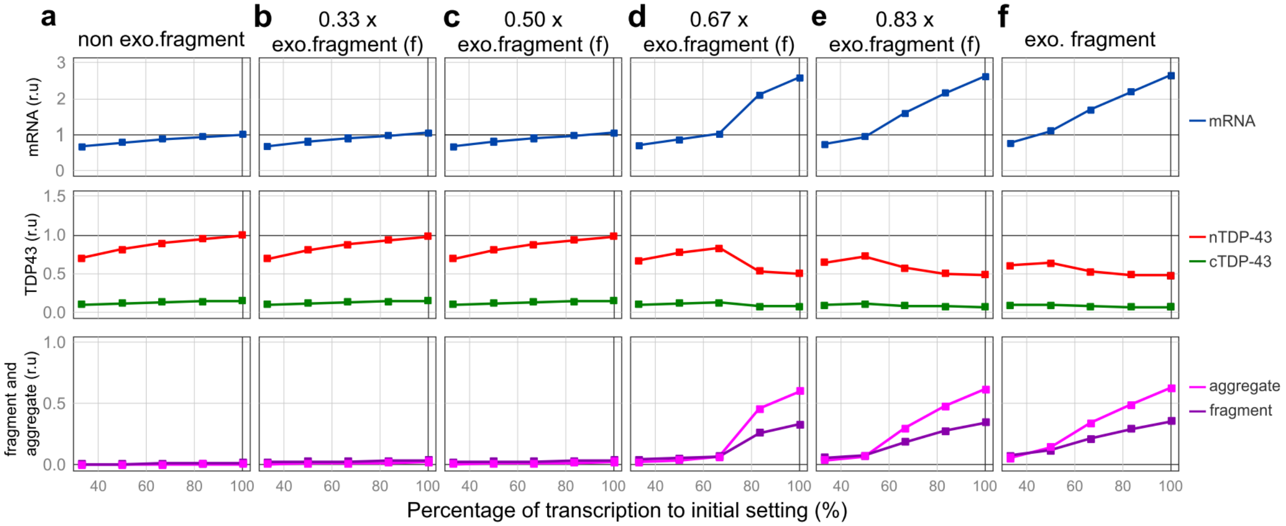

Supplementary Figure 6. Therapeutic effects on TDP-43 propagation (Supplement to Figure 6D-E). Under the condition in which different amounts of extracellular fragments are assumed **(a-f)**, the change in each element related to a decrease in transcription is shown.
